# Miscellanea

**Published:** 1855-10

**Authors:** 


### MISCELLANEA.

*Editorial Changes.*—Dr. H. D. Bulkley has resumed the editorship of the New York Medical Times.

*Female Doctors.*—Helen M. Gassett, of Boston, who acted for a time as one of the agents for raising funds in behalf of the N. E. Female Medical School, is out in a pamphlet which purports to be an exposition of the dishonesty and frauds of some of the Directors of the college. Mrs. Gassett charges them with being thieves and humbugs.

*Gambling to the Teeth.*—A Texas paper relates the following incident as having recently occurred in the "Lone Star:" "An individual, a few nights ago, being put to for a stake, took from his mouth a finely polished set of teeth on a gold plate, and pawned it for a few dollars to continue the game with." We have heard of "fighting to the teeth," but never before heard of gambling to the teeth.

*Heavy Fees.*—Dr. Dimsdale, of Hertford, visited Russia to inoculate the Empress Catherine and her son, for which service he received the rank of Baron of the Empire, &c., besides a pension of £500 per annum, and a present of £12,000.—Dupuytren made his last professional visit to one of the Rothschilds, who gave him 100,000 francs for attending to his fractured femur.—Sir Astley Cooper, after performing the operation of stone upon Mr. Hyatt, a West Indian merchant, was presented with a fee of 1000 guineas.

*The Income of Sir Astley Cooper in Early Life.*—"My receipts," says he, "for the first year were £5 5s.; the second £26; the third £64; the fourth £95; the fifth £100; the sixth £200; the seventh £400; the eighth £600; the ninth £1100."

Dr. Pond, of Rutland, N., has invented a pill-making machine, which manufactures any quantity of those articles in a day.

Of twelve American surgeons who have gone out to join the belligerent armies in Europe, nine have attached themselves to the service of the Czar, and three have attached themselves to the Allies.

London papers announce the death of Dr. Archibald Arnott, in the eighty-fourth year of his age. He was Napoleon's last medical attendant. Napoleon expired with his right hand in that of Dr. Arnott.

The Gazette Medicale complains of the difficulty experienced in obtaining a sufficient number of military surgeons for the armies in the East.

During the months of January and February, according to the Inspector General's Report, there were issued by the Purveyor to the Forces in the Crimea, 560 doz. port wine; 131 doz. sherry wine; 630 bottles rum; 8 gall. rum; 688 bottles brandy; 1724℔ tea; 7920℔ sugar; 2111℔ preserved meat, &c., &c.,

Wong Tun is the only native of China who has ever received the honor of medical graduation in Edinburgh.

*Turkish Order of Knighthood,*—Dr. C. T. Jackson received by the last steamer from Europe the patent and decoration of the Order of Knighthood conferred upon him by the Sultan of Turkey, Abdul Medjid, for services rendered by his discovery of Anæsthesia to the armies of the Allies. This is one of the fifteen decorations of this order lately conferred by the Sultan on those who have rendered service to the empire.

*Change.*—Prof. Torrey has resigned the chair of Chemistry in the College of Physicians and Surgeons of New York, and Dr. Leconte, of Georgia, has been appointed in his place.

Dr. P. Claiborne Gooch, of Richmond, Va., the founder of the Stethescope, recently fell a victim to Yellow Fever in Norfolk, where he had gone on the humane mission of affording succor to the distressed population of that city.

*Statistics of Paris.*—The population of Paris may be considered, with the environs, as 1,200,000. The average daily consumption of bread in the city is a million of pounds, or a pound for each person; but, as every workman eats three pounds a day, it is found that the consumption by women, by children, and by the aged, which is considerably under a pound a-piece, furnishes an ample compensation. The climate is calculated to require a person in good health to consume one pound of meat, one and a half of vegetables, and one and a half of bread, with a bottle of claret, or two bottles of beer. The consumption of bread diminishes in years of abundant wine yields, and *vice versa.* A heavy rise in the price of bread increases the number of deaths very perceptibly. The 40,000 cats and 70,000 dogs of the city of Paris, eat six millions pounds of bread a year. Unwise economists have proposed their destruction, in view of the saving that might be effected ; but it is clear that it would only provide for six days' consumption out of three hundred and sixty-five. There are 601 bakers in Paris, who are divided into four classes :—the first, including those who use more than four bags of flour a day ; the second, third and fourth, those who use three, two, and less than two a day. A bag contains 314 pounds of flour, and furnishes 408 pounds of bread. Any baker who puts more water into a bag full than is necessary to raise it up to this standard weight ; or any one who adulterates his flour with mixtures of carbonate of magnesia, bi-carbonate of soda, or powdered alabaster, is punished by a fine of thirty francs and a week's imprisonment. Frauds in bread, however, are extremely rare. There are 500 butchers in the city. The number of persons in Paris who abstain entirely from meat, is so large, that the average consumption of the city is reduced to three ounces a day for each person. It is the class that goes without meat that fills the hospitals. The average wine yield of France is 1,000,000,000 gallons—two-thirds of which is consumed in the country, and one-third exported. Of the 333,000,000 gallons annually drank in France, Paris claims 40,000,000—that is five times as much as its proportion of population entitles it to. Of this quantity, 7,000,000, or one-sixth, are supposed to be added, either in the form of water, or of decoctions purely artificial. The government does what it can to punish and prevent frauds of this sort, and it keeps in its employ sworn and patented tasters, whose palates possess a sort of humming-bird delicacy.

